# Value of Newborn Hearing Screening on Early Intervention in the Saudi Population and Review of International Records

**DOI:** 10.7759/cureus.5990

**Published:** 2019-10-24

**Authors:** Yazeed A Alshawi, Najd Al-Gazlan, Fahad Alrawaf, Fida Almuhawas

**Affiliations:** 1 Otorhinolaryngology Head and Neck, Prince Sultan Military Medical Hospital, Riyadh, SAU; 2 Otorhinolaryngology Head and Neck, King Saud University, Riyadh, SAU; 3 Otolaryngology, King Abdullah Ear Specialist Center, King Saud University, Riyadh, SAU

**Keywords:** delayed presentation, deaf, cochlear implant (ci), sensorineural hearing loss (snhl), newborn hearing screening, family history, universal newborn hearing screening (unhs)

## Abstract

Background

Hearing impairment is found to be the most prevalent disabling condition worldwide. Early diagnosis is crucial to avoid speech and language delays and to ensure the best performance results after cochlear implant (CI) surgery. Universal newborn hearing screening is a way to recognize newborns with a hearing impairment with or without risk factors. In this article, we have studied the effect of the newborn hearing screening program on early presentation to a healthcare center and, hence, early intervention in patients with congenital hearing loss, and reviewed the international numbers.

Objectives

The objective of this study was to determine whether neonatal hearing screening in Saudi Arabia helped prelingually deaf children to present earlier or not.

Design

Retrospective cross-sectional review

Setting

King Abdullah Ear Specialist Center (KAESC), Riyadh, Saudi Arabia

Subjects and methods

We included all patients who presented to the CI committee for the first time at KAESC, between March 2016 and March 2018, and met the inclusion criteria. Data were retrieved through phone calls and patient files. The sample size was 242.

Main outcomes

The timing difference between those who were screened positive for hearing loss at birth versus patients who were screened negative or not screened at all.

Results

By far, patients who were screened positive for hearing loss presented earlier (p-value >0.001) to a healthcare center than those who were not screened at all or screened negative for hearing loss and they finished the journey to CI 17 months earlier than those who were not screened. On the other hand, those who were screened negative were not found to present later than those who were not screened.

Conclusion

Going with the international trend, screening was found to have a significant positive effect on age at presentation, diagnosis, hearing aid fitting, surgery, and, hence, performance after implantation. Testing false negative on screening did not show a significant further delay when compared to those who were not screened.

## Introduction

Hearing impairment is considered the most prevalent disabling condition worldwide [1]. According to the World Health Organization (WHO), close to 360 million individuals are affected by debilitating hearing impairment, with 32 million being children [1]. Approximately two-thirds of individuals with hearing impairment live in developing countries, where 2000 neonates with impaired hearing are born daily [1]. Hearing loss can be conductive, sensorineural, mixed, or central [2]. The crucial period for auditory development in humans starts around the twentieth week of gestation and continues until three years of age [3]. Genetic and environmental factors are recognized causes of hearing impairment [3]. Children with this disability face difficulties related to language development, speech, emotions, and social behavior [3]. At present, transient evoked otoacoustic emission (OAE) and automated auditory brainstem response (aABR) are the predominantly used technologies for hearing screening, with aABR known to exhibit high sensitivity and specificity, a low referral rate, and a low false-positive rate [3]. OAE tests are also used to screen newborns for congenital hearing loss in several universal newborn hearing screening (UNHS) programs; however, they are associated with high false-positive and referral rates [4]. Repeat tests and advanced age at the time of initial screening are considered to lower the referral rate [4]. In other words, delayed newborn hearing screening can result in better hearing outcomes [4]. Moreover, the use of higher frequencies and more advanced OAE devices is recommended for better results in newborn hearing screening [4]. The common risk factors for congenital hearing loss include premature birth requiring neonatal intensive care unit admission and cytomegalovirus infection [2]. However, as many as 42% of children with a profound hearing impairment remain undiagnosed by risk-based screening alone [2]. A previous study demonstrated that parents often experienced difficulties in detecting the effects of hearing impairment through observation of their neonates at home, particularly in cases of mild or moderate impairment [5]. The authors concluded that delays in the fitting of hearing aids could be attributed to the lack of parental knowledge and awareness of the hearing difficulties experienced by the infants and that further parental support should be provided in order to improve their understanding [5]. UNHS is a way to recognize newborns with hearing impairment, regardless of the presence of risk factors [2]. Newborns with positive screening tests need intervention services and referral for definitive testing [2]. This study is the first of its kind in Saudi Arabia with the purpose of determining whether neonatal hearing screening in Saudi Arabia helped prelingually deaf children to present earlier or not.

## Materials and methods

Aim of the study

In the present study, we evaluated children with and without neonatal hearing screening in Saudi Arabia and determined the mean ages at the time of the following events: suspicion of hearing loss, presentation to the hospital for audiological evaluation, diagnosis, hearing aid fitting, and presentation to the cochlear implant (CI) committee. We also evaluated if patients with congenital hearing loss who showed negative results in initial screening tests, which could be due to false-positive aABR test findings or auditory neuropathy patients who were screened with OAE, presented later than those who were not screened at all. Finally, we aimed to gather a better understanding of the potential causes of delays in audiological testing and intervention in all evaluated children.

Study design and setting

This study was a retrospective cross-sectional study, which was conducted at a tertiary hospital (King Abdullah Ear Specialist Center (KAESC) at King Abdulaziz University Hospital) in Riyadh, Saudi Arabia.

Study participants

Inclusion Criteria

All pediatric patients (up to the age of 18 years) with bilateral severe to profound hearing loss and prelingual deafness who presented to the CI committee for the first time.

Exclusion Criteria

All adult patients who presented for second ear CI, patients with post-lingual deafness, patients with acquired hearing loss, and any patients with a psychological or neurological condition.

Sample Size

The sample size of this study was 242 patients who fulfilled our inclusion and exclusion criteria.

Sampling Technique

The participants were recruited using convenient sampling. We included all patients who presented to the CI committee for the first time at KAESC, between March 2016 and March 2018.

Data collection methods, instruments used, and measurements

Each patient with a sensorineural hearing loss who has been referred to the CI committee at KAESC gets registered and undergoes a clinical evaluation and different investigations, which are all kept in an electronic file in KAESC’s registry. The patients were recruited from the CI committee registry and then our inclusion and exclusion criteria were applied. The data were collected using a self-designed data collection sheet, which consisted of two subdivisions: the first one examined the socio-demographic characteristics of the participants (i.e. gender, age, whether the patient had a pre or post-lingual deafness, status of the other ear, and medical status) while the second part examined the ages of the patients by months at suspicion of hearing loss, diagnosis, hearing aid fitting, presentation to the CI committee and whether the patient was screened at birth or not. Data were collected using phone calls with parents or a direct caregiver (for those whose parents are deceased) and patient files. The data were then transferred to an Excel sheet. Patients who were screened positive for hearing loss at birth were compared with patients who were screened negative at birth for hearing loss or those who were not screened at all in terms of age (months) at suspicion, first audiological testing, a confirmed diagnosis, magnification, and presentation to the CI committee.

Data management and analysis

Statistical tests were carried out using Statistical Package for the Social Science (SPSS) software (version 26; IBM Corp., Armonk, NY, US). The analysis of variance (ANOVA) test was used to compare the study groups. The post-hoc test was applied to compare the groups with each other. A p-value of less than 0.05 was considered significant.

Ethical consideration

The research received institutional review board (IRB) approval at King Saud University Medical City (KSUMC). Confidentiality was maintained. The research was fully explained to the patient's relatives, and verbal informed consent was obtained from them during the phone call. Patient relatives were informed that they were free to withdraw at any time and that would not affect their clinical treatment.

## Results

**Figure 1:**
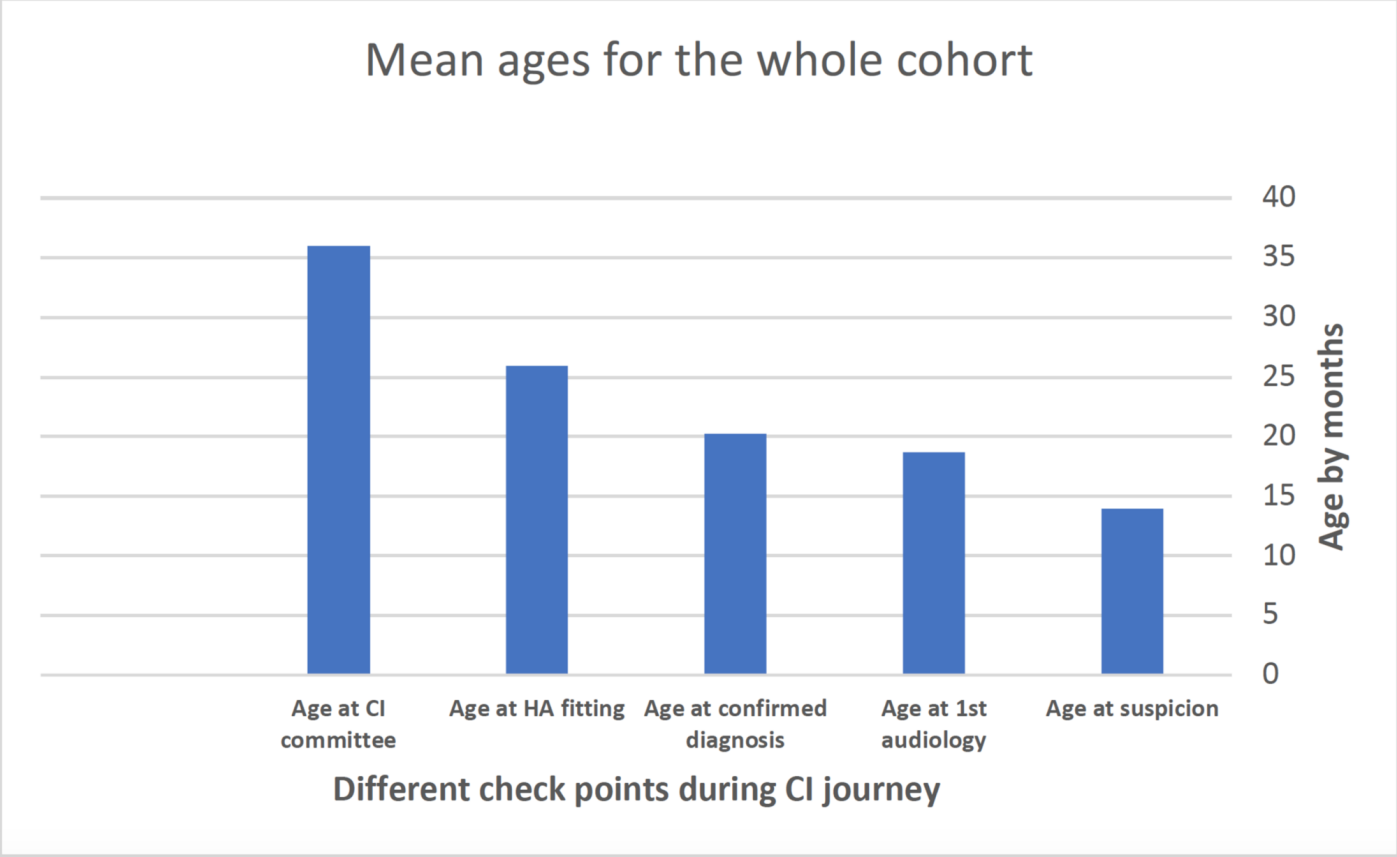
The mean ages by months at the different checkpoints during the journey to CI for the overall sample CI: cochlear implant

Among the 242 patients, only 23 (9.5%) were screened positive for hearing loss at the neonatal hearing screening (did not pass the hearing screening) while 37 (15.3%) were screened negative at the newborn hearing screening (passing the hearing screening ) and 182 (75.2%) were not screened at birth. The mean ages (months) at the time of the following events for the overall cohort are shown in Figure 1: suspicion of hearing loss, first audiological test, diagnosis, hearing aid fitting, and presentation to the CI committee.

The mean ages at the same checkpoints for the three individual groups are shown in Table 1.

**Table 1:** The mean ages by months for the three individual groups HL: hearing loss, CI: cochlear implant

		Number of patients	Mean age by months	Standard Deviation	Standard Error
Suspicion of hearing loss	Screened positive for HL	23	2.39	3.677	0.767
	Screened negative for HL	37	14.95	10.371	1.705
	Not screened at all	182	15.23	14.645	1.086
	Total	242	13.97	13.875	0.892
First audiological test	Screened positive for HL	23	4.65	5.890	1.228
	Screened negative for HL	37	18.65	12.074	1.985
	Not screened at all	182	20.48	17.580	1.303
	Total	242	18.69	16.682	1.072
Diagnosis	Screened positive for HL	23	5.78	7.367	1.536
	Screened negative for HL	37	19.46	12.105	1.990
	Not screened at all	182	22.23	18.343	1.360
	Total	242	20.24	17.394	1.118
Hearing aid fitting	Screened positive for HL	23	15.87	14.166	2.954
	Screened negative for HL	37	25.22	13.697	2.252
	Not screened at all	182	27.37	19.778	1.466
	Total	242	25.95	18.747	1.205
Presentation to the CI committee	Screened positive for HL	23	20.00	16.561	3.453
	Screened negative for HL	37	39.84	23.307	3.832
	Not screened at all	182	37.26	27.780	2.059
	Total	242	36.02	26.715	1.717

The ANOVA test revealed significant differences in all mean ages among the three groups (suspicion: p-value = 0.000, first audiological test: p-value = 0.000, diagnosis: p-value = 0.000, hearing aid fitting: p-value = 0.020, presentation to the CI committee: p-value =0.009). Therefore, the post-hoc test was done to compare them with each other. The results are shown in Table 2.

**Table 2:** Comparisons of mean ages (months) at the suspicion of hearing loss, first audiological test, diagnosis, hearing aid fitting, and decision for CI in patients with severe to profound hearing loss who showed positive or negative findings in newborn hearing screening and patients who were not screened at birth *p-value < 0.05 is considered statistically significant CI: cochlear implant

Dependent variables			Mean difference	Standard error	p-value*
Suspicion of hearing loss	screened positive	screened negative	−12.555	1.869	0.00
		not screened	−12.839	1.329	0.00
	screened negative	not screened	−0.258	2.021	0.989
First audiological test	screened positive	screened negative	−13.966	2.334	0.00
		not screened	−15.862	1.791	0.00
	screened negative	not screened	−1.829	2.375	0.722
Diagnosis	screened positive	screened negative	−13.677	2.514	0.00
		not screened	−16.443	2.051	0.00
	screened negative	not screened	−2.766	2.410	0.488
Hearing aid fitting	screened positive	screened negative	−9.347	3.714	0.040
		not screened	−11.499	3.298	0.004
	screened negative	not screened	−2.152	2.687	0.704
Presentation to the CI committee	screened positive	screened negative	−19.838	5.158	0.001
		not screened	−17.264	4.021	0.000
	screened negative	not screened	2.574	4.350	0.825

As shown in Table 2, the families of patients who showed positive screening findings for hearing loss suspected that their children had a serious hearing problem at a mean age of 2.4 months (although some of them were reassured that the tests were only preliminary screening tests) while patients with a negative screening test (passed the hearing screening tests) and patients who were not screened at birth were suspected to have hearing loss 12.1 and 12.8 months later, respectively. Consequently, patients with positive screening findings for hearing loss presented to the hospital for a hearing assessment at a mean age of 4.7 months, which was 13.3 and 15.9 months earlier than the mean ages at which patients with negative screening findings and patients without screening, respectively, underwent their first audiological evaluations. At a mean age of 5.9 months, patients with positive screening findings had a confirmed diagnosis of hearing loss, and they were fitted with hearing aids at a mean age of 15.7 months. In comparison, patients with negative screening findings were 12.9 and 8.8 months older at the time of diagnosis and hearing aid fitting, respectively, while those without screening at birth were 16.4 and 11.8 months older, respectively.

When comparing patients who were screened negative and those who not screened at birth, patients with negative screening findings and those who were not screened were suspected by their families to have a hearing loss at the mean ages of 14.5 and 15.2 months, respectively; thus, there was no significant difference between the two groups. The first audiological test in the negative screening group was performed 2.5 months earlier (mean age: 17.9 months) than that in the no screening group; this difference was also not significant. In the negative screening group, the diagnosis was confirmed at a mean age of 18.8 months, hearing aids were fitted at a mean age of 24.5 months, and presentation to the CI committee was at a mean age of 27.6 months. These values were 12.3,39.4, and 37.2 months, respectively, for the no screening group, with no significant differences between the two groups.

## Discussion

Severe to profound hearing loss children who undergo newborn hearing screening at birth are much more likely to be diagnosed and treated in a timely manner. Newborn hearing screening programs aim to lower the ages at the time of audiological testing and intervention for children with hearing impairment. Early detection and intervention for prelingual bilateral severe to profound hearing loss are expected to minimize delays in language development. Several nations have begun implementing early identification and interventions for hearing loss and are conducting regional UNHS programs. In a study conducted in Champagne-Ardenne, hearing impairment was diagnosed at an average age of 3.2 months, and confirmation of the initial diagnosis in most children was facilitated by follow-up auditory examinations [6]. The authors mentioned that various auditory interventions can be undertaken depending on the degree of hearing loss [6]. Another study in Paris recruited 27,885 newborns and found that the mean age at the time of hearing aid fitting ranged from four months for children with profound hearing loss to 11.4 months for those with moderate hearing loss [7]. The mean age at cochlear implantation was 14 months [7]. In a study in Los Angeles, hearing loss was diagnosed 24.62 months earlier, hearing aids were fitted 23.51 months earlier and intervention was performed 19.98 months earlier for children who were screened as newborns than for those who were not screened [8]. In Italy, the mean age at diagnosis of severe to profound hearing loss was 20.5 months for the entire cohort, 6.8 months for children who were screened as newborns, and 29.3 months for those who were not screened [9]. As a result of successful UNHS programs in Philadelphia, the age at diagnosis decreased from two years to three to six months [10]. Moreover, speech and language outcomes were better for children diagnosed with hearing loss before speech development than for children with a delayed diagnosis [10]. In a New York study, the median age at the identification of hearing loss and early intervention was three months while that at hearing aid fitting was 7.5 months [11]. In Austria, hearing loss was identified at a mean age of 3.9 months in children who underwent UNHS and 37.6 months in those without screening [12]. By the age of six months and one year, 69% and 80% of infants who underwent screening, respectively, were identified to have hearing loss; these values were only 6% and 12% for infants without screening [12]. The degree of hearing loss in children without screening was the strongest predictor of the age at diagnosis (median age at the diagnosis of profound hearing loss, 15 months; severe hearing loss, 26 months; moderate hearing loss, 52 months; mild hearing loss, 73 months) [12]. On the other hand, there was no association between the age at diagnosis and the degree of hearing loss in children who underwent UNHS (median age: 3.7 to 4.4 months) [12]. Another Austrian study reported that 35% and 2% of children with and without UNHS, respectively, were diagnosed with hearing loss at the age of three months [13]. These percentages increased to 69% and 6%, respectively, at six months of age and 81% and 12%, respectively, at one year of age [13]. At six months of age, 61% of children who were screened and only 4% of children who were not screened had received treatment [13]. A study conducted from 2006 to 2011 in Belgium reported that the hearing screening program in Wallonia and Brussels promoted earlier audiological intervention among hearing-impaired children [14]. In Thailand, children with congenital hearing loss, which was detected by screening were diagnosed and treated by six months of age [15]. In Flanders and The Netherlands, children who were screened early were significantly younger than those with a delayed screening at the time of the diagnosis of hearing loss and cochlear implantation [16]. Moreover, early screening was associated with better receptive and expressive spoken language skills after cochlear implantation [16]. In Saudi Arabia, the Ministry of Health (MOH) has launched the first phase of national newborn screening for hearing loss in late 2016. Before that, some hospitals (mainly military medical centers, specialized pediatric centers, and some private hospitals) have implemented newborn hearing screening in their own protocols and most of our patients are a product of these programs.

In the present study, hearing loss was suspected much earlier in children who showed positive findings for hearing loss in newborn hearing screening than children who passed the screening test (either due to false-positive aABR test findings or auditory neuropathy patients who were screened with OAE) and children who were not screened at birth. As a result, the mean ages at the first audiological test, diagnosis, and hearing aid fitting were much lower for children with positive findings than for those with negative findings and those without screening. On the other hand, children with negative screening findings and those without screening showed no significant differences with regard to the mean ages at the suspicion of hearing loss, first audiological test, diagnosis, hearing aid fitting, and presentation to the CI committee. These findings suggest that even though families of children with hearing loss who passed the screening test were reassured that their children are hearing, they did not present later than those who were not screened at all.

We also observed significant delays in audiological testing and hearing aid fitting in children with positive screening findings at birth. While audiological testing was performed 1.7 months later than the age of three months recommended by the Joint Committee on Infant Hearing (JCIH) 2007, hearing aids were fitted around 10.1 months later than the recommended age (within one month after diagnosis) [17]. Since access to healthcare services is critical to good health, a variety of access barriers could be faced. For instance, some government hospitals may lack the availability of hearing aids at a particular time either due to the location of the hospital or other various causes, which finally will lead to this unnecessary delay.

A study conducted in Taiwan showed that UNHS helps in the early detection, diagnosis, and treatment of congenital hearing loss [18]. In that study, the average age at diagnosis was 8.7 months, that at hearing aid fitting was 12.4 months, and that at auditory intervention was 18.8 months for children who underwent newborn screening [18]. These ages were 27.5, 31.3, and 40.5 months, respectively, for children who were not screened [18]. Thus, similar to the findings in the present study, there were significant differences in the ages at diagnosis, hearing aid fitting, and auditory intervention between children who received UNHS and those who did not [18]. These findings, together with ours and others mentioned above, suggest that the endorsement and strict implementation of UNHS is gradually lowering the ages at diagnosis and treatment of hearing loss in children. Negative screening findings didn’t show significant differences when compared to those patients who weren’t screened at all, which contributed to limiting the early diagnosis and treatment of children. We can prevent them by screening patients with aABR, not only OAE, repeating the screening for patients whose test was inconclusive or with unclear results. Low sample size, especially for those who were screened and for those with negative screening findings where we couldn’t tell if they were screened with OAE or aABR, was the limitations of our study. Yet, this is the first paper about neonatal hearing screening in Saudi Arabia.

## Conclusions

Consistent with international trends, the findings of our study in Saudi Arabia showed that children with severe to profound hearing loss who undergo newborn hearing screening at birth are significantly more likely to be diagnosed and treated in a timely manner. However, negative screening findings due to reasons such as false-positive ABR test findings and auditory neuropathy (in patients who were screened with only OAE) can have the same effects as a complete lack of screening. Further studies, including larger sample sizes and different speech tests, are necessary to understand the effects of UNHS on CI performance.
